# Psychometric testing of a checklist for procedural training of peripheral intravenous insertion

**DOI:** 10.1186/s41077-019-0092-y

**Published:** 2019-04-18

**Authors:** Lisa A. Buckley, Gregory E. Gilbert, Eric B. Bauman

**Affiliations:** 1 Ross University School of Medicine, 2300 SW 145th, Suite 200, Miramar, FL 33027 USA; 2 SigmaStats© Consulting, LLC, 1865 Bairds Cove, Charleston, SC 29414 USA; 3 Clinical Playground, LLC, 1615 Maple Street, Middleton, WI 53562 USA

**Keywords:** Checklist, Clinical skills, IV insertion, Venous cannulation, Reliability, Validity, Generalizability theory, Low-fidelity simulation, Simulation

## Abstract

**Background:**

Nurses, medical technologists, nuclear medicine technologists, pre-hospital providers, and medical students are a few groups of healthcare learners asked to learn intravenous (IV) cannulation in their training (J Surg Educ. 69:536–43, 2012). Despite the fact that IV cannulation has been taught to several health professions, it is difficult to find a psychometrically validated checklist to guide teaching this skill in the simulated procedural training (Pediatrics 124: 610-9, 2009, J Assoc Vasc Access 21: 196-204, 2016). In the absence of a pragmatic, valid checklist for the initial teaching of peripheral IV skills in the simulation procedural skills lab, this investigation sought to describe the process and create a psychometrically valid checklist.

**Methods:**

Expert raters used Lawshe’s method for identifying valid items from the universe of items for IV insertion. Gwet’s AC_2_ and generalizability (G) theory was used assess inter-rater reliability.

**Results:**

The literature and in-house IV checklists were examined for steps to inserting a peripheral IV, and the steps were compiled into a survey and sent to experts who rated each item. Of the 37 potential steps, 16 steps were identified as being psychometrically valid. The checklist content validity index was .82. Inter-rater reliability was .94 (95% CI .91–.98). Good inter-rater reliability was confirmed using generalizability theory.

**Conclusions:**

This study created and provided evidence of content validity and reliability for this checklist using Lawshe’s methodology. As such, this method of evaluating a checklist for validity and reliability evidence can be followed for other healthcare checklists. This checklist can be used for teaching IV placement in healthcare students in the simulation procedural training lab.

**Electronic supplementary material:**

The online version of this article (10.1186/s41077-019-0092-y) contains supplementary material, which is available to authorized users.

## Introduction

Despite the fact that intravenous (IV) cannulation has been taught to several health professions, it is difficult to find a standardized checklist with evidence of psychometric validity for teaching this motor skill in the simulation procedural skills lab [2–5]. Nurses, medical technologists, nuclear medicine technologists, pre-hospital responders, and medical students are a few groups of healthcare learners asked to learn IV cannulation in their training [1]. Intravenous catheters are often placed in real patients by healthcare students and workers who have little education, training, and opportunities for practicing the skill [3]. As a result there are more failure rates leading to patient discomfort, prolonged time-to-treatment, and increased healthcare costs [3, 6].

The intent of this paper is to develop and describe the process of developing a pragmatic checklist with psychometric validity to teach healthcare professionals IV cannulation as part of simulation procedural training. Psychometric validity is the degree to which a test measures what it is supposed to measure [7]. Despite several commonly used checklists, the ability to find a checklist with documented psychometric validity is challenging. Most published checklists do not specify the methodology used to develop the checklist.

### Conceptual model

Venous cannulation competency is broken into several components: motor skill, confidence, communication skills, understanding and applying concepts of anatomy and physiology, understanding indications, contraindications, and complications of the procedure [8]. The procedure fits well within Gagne’s model of instructional design which is based on the mental information-processing model adult learners progress through when learning new skills [9]. The model includes nine instructional events, all of which must be present, in the learning process: gaining attention, informing learner of objectives, stimulation recall of prior learning, presenting the stimulus material, providing learning guidance, eliciting the performance, providing feedback about correctness of the performance, assessing performance, and enhancing retention and transfer of knowledge and skills [9].

The authors chose a pragmatic standpoint due to their own anecdotal experience of training a high volume of learners in the IV placement procedure in a hospital-based simulation lab with real-world constraints [10]. The main constraints include limited availability of trained faculty instructors, simulation lab supplies, and lab availability. The use of a 25- to 30-step checklist was not practical within these constraints; therefore, the authors set out to validate a checklist that could be used in the simulation procedural training of motor skills.

## Methods

### Checklist item search

The checklist was developed by beginning with a literature search of PubMed, AccessMedicine, and UpToDate and asking for in-house versions of IV checklists from our nursing and medicine colleagues. PubMed was searched for occurrences of “intravenous cannulation checklist.” AccessMedicine and UpToDate were searched for “intravenous cannulation.”

### Validity

The contemporary definition of validity is, “… the degree to which evidence and theory support the interpretations of test scores entailed by proposed uses” of a test [11]. The American Educational Research Association, American Psychological Association, and National Council for Measurement in Education have jointly authored the *Standards for Educational and Psychological Testing* [11]. The *Standards* put forth the ideal to which all testing should subscribe. The *Standards* outline five types of evidence for psychometric instrument interpretation: test content, internal structure of the test, response processes, association with other variables, and consequences of testing. Evidence of test content validity can be seen when the content of an instrument matches the content that should be included in the test. Evidence of good internal structure of an instrument is assessed using coefficient alpha also known as tau-equivalent reliability [12, 13]. Response processes validity evidence is how participants approach solving a problem. Does the instrument mimic how the process takes place? Validity evidence is reflected in association with other variables when the same participants perform similarly on other, congruent instruments. Finally, validity evidence through consequences of testing refers to policy or performance outcomes after an instrument has been implemented. This study only dealt with creating an instrument with content validity evidence.

To establish the content validity of a psychometric instrument Lawshe suggests asking experts the question “Is this essential; useful, but not essential; or not necessary … ?” As an example, an item would ask “Is it essential; useful, but not essential; or not necessary to don gloves?” An expert for this study was defined as an individual who as part of their routine clinical work frequently performs IV placement. Responses are then weighted and the content validity ratio (CVR) calculated [14]. The content validity ratio is a measure of the percentage of panelists agreeing an item is essential. Items equal to or greater than the CVR critical value are kept. A simplified table of CVR critical values, which corrected a mistake in Lawshe’s original table, was used for inclusion of checklist items [15]. The content validity index was calculated, which is the average of the remaining content validity ratios [14]. The full survey sent to the experts is available in Additional file 1, and each expert was instructed to rate each item based on performing the procedure on a patient.

### Inter-rater reliability

Gwet’s AC_2_ was used to assess inter-rater reliability [16, 17]. There is a well-documented paradox of low-reliability in the presence of high inter-rater agreement, and Gwet’s AC overcomes this paradox [18–20]. Reliability was also assessed using generalizability theory (G theory) [21]. G theory can be thought of as a multidimensional way of looking at reliability. Traditional test theory can only examine one type of reliability at a time and one “factor” (called facets in G theory) at a time, G theory allows researchers to consider many more. G theory allows researchers to not only generate an analog to traditional reliability coefficients (the G and phi coefficients), but allows researchers to isolate the variance each facet contributes to the study. With G theory, researchers can go further and examine if raters are ranking students (or items) in a similar fashion. G theory provides researchers with more information to better inform decisions regarding assessments. Two very approachable introductions to G theory are Prion et al. and Bloch and Norman [22, 23].

The G coefficient quantified how consistently raters agree on the usefulness of the items and expresses the degree to which observed differences among the raters are consistent with differences that would be obtained if nearly an unlimited number of observations were obtained.

### Analysis

A survey of the universe of ordered items pertaining to IV insertion was assembled and made available to paramedics and registered nurses including certified nurse anesthetists. Data were entered into a Microsoft Excel (Redmond, WA) spreadsheet according to guidelines outlined in Broman and Woo [24] and saved in a comma-separated value (CSV) format. To obtain what was felt to be the most robust checklist, a CVR and CVI were calculated for all data. Items not meeting the critical values established by Ayre and Scally [15] were deleted. Inter-rater reliability was assessed using two measures: Gwet’s AC_2_ reliability coefficient and G theory [21] using GENOVA (Iowa City, IA) software. All analysis, except the G study, were done using R software v3.3.1 (Vienna, AT).

## Results

### Checklist item search

The PubMed search revealed no occurrences of checklist, AccessMedicine revealed one checklist (Tintinalli), and UpToDate revealed one checklist (Frank) [25, 26]. Consultation with colleagues in key curricular leadership positions from medicine and nursing uncovered one checklist from medicine and three from nursing (Ross University School of Medicine. Intravenous Cannulation Checklist. Miramar, FL: Unpublished; 2016). These included Wilkinson and Van Lauven’s checklist from *Procedure Checklists for Fundamentals of Nursing*, *Infusion Nursing Society*, and *Thomson Delmar Learning* [27–29]. Despite locating the six intravenous cannulation insertion checklists, no evidence of psychometric validity was found [2, 4]. The steps were extracted from each checklist and similar steps were combined.

### Validity

After the items were compiled and ordered, they were assembled into a survey subscribing to Lawshe’s methodology (Additional file 1). The survey was distributed to paramedics in a Midwestern state via an email listserv and to registered nurses in two academic hospitals, one located in a Midwestern state and one located in the Northwest. Eleven paramedics and 13 registered nurses, including Certified Registered Nurse Anesthetists (CRNA) responded. The median number of years of experience was 10 (IQR 9.8).

In accordance with Lawshe’s methodology, items less than the CVR critical value were removed from the checklist [14]. Non-essential items were examined. The removed items include the following: tape, IV connector, removing the Luer adapter/cap, keep flush attached, placing the tourniquet proximal to insertion site tight enough to occlude venous flow, palpating for veins that appear easier to cannulate distally on the non-dominant arm, cleaning the area circumferentially from center to periphery, not touching the part of the catheter that will be inside the patient’s arm/vein, applying distal traction by placing your hand away from the needle insertion site, when there is blood return lowering the angle of the needle to 10°, removing the tourniquet, connecting an IV tube extender, drawing back on the plunger to check for blood return, reconnect the Luer adapter, tape the tubing in place with two separate pieces of tape, and disposing of the needle in the sharps container. Although these steps may be taught as part of the procedure, the experts did not feel they were essential to accomplish intravenous cannulation. The final checklist can be seen in Additional file 2: Figure S1.

### Reliability

Inter-rater reliability was assessed for the entire group and for nurses (CRNAs included) and paramedics separately. Gwet’s AC_2_ for the entire group was .94 (95% CI .91–.98). Nurses demonstrated an inter-rater reliability of .94 (95% CI .87–1.00) while paramedics had a slightly lower inter-rater reliability (.92, 95% CI .84–.99). All inter-reliability coefficients can be considered high.

Seven percent of the variance was attributed to the raters (nurses, CRNAs, and paramedics), 0% of the variance attributed to the items, and 93% of the variance attributed to the interaction between the two and residual variance. Seven percent of the variance attributed to expert raters can be interpreted as good inter-rater reliability correlating with Gwet’s AC_2_. Most of the variance was found in the interaction and residual error term suggesting a substantial amount of variance unaccounted for by the raters or the items. The overall G coefficient for the instrument was moderate (*G* = .52), indicating the instrument provides consistent information across the universe of raters.

## Discussion

Validity is a concept that is context dependent, and therefore, we cannot claim that this checklist is valid in all situations. Rather, this checklist has demonstrated content validity and reliability through this study. This means that it can be used for teaching IV cannulation to healthcare students, and it includes the essential steps to accomplishing this procedure in patient care according to our expert panel.

Intravenous placement competency includes several components: motor skill, confidence, communication skills, understanding and applying concepts of anatomy and physiology, understanding indications, contraindications, and complications of the procedure [8]. The checklist focused on the motor skills involved in the insertion of an IV line and applying concepts of anatomy and physiology specifically for use in simulation procedural training. The developed checklist emphasizes the three components of Gagne’s model for instruction which can be realistically accomplished in the simulation procedural training lab. This checklist should be used as part of a more comprehensive IV training curriculum and does not represent a gold standard or a full curriculum for teaching this skill. Rather, the checklist can be viewed as a piece of quantifiable evidence in the process of preparing healthcare students for IV cannulation during simulation-based learning exercises prior to subsequent clinical practice [3].

This study has several limitations. The first limitation is the lack of healthcare provider responses other than paramedics, nurses, and CRNAs, which could cause an unknown sampling bias. Replication of this study using respondents who are IV team experts would strengthen the evidence. Another limitation of this study is that it is focused on three of the nine steps in Gagne’s instructional model, which means we did not create or propose a thorough teaching strategy for IV cannulation. However, the purpose of this study was to provide psychometrically validated evidence for an IV placement checklist. Another limitation of this study is that all supplies and terms used on the checklist may not be universal or available in all resource settings.

These results provide a solid foundation and framework for further research on validation of procedural skills checklists. It is not uncommon in healthcare to use teaching and assessment tools that have not been evaluated for psychometric validity. This approach using Lawshe’s method can be used to evaluate other checklists for educational skills and processes found in healthcare for validity evidence. Several future research questions remain: how do student learning outcomes compare when using this checklist against a current method of teaching IV cannulation? How often do learners need to review the checklist steps in order to maintain proficiency of IV placement skills? Future studies should evaluate the use of this checklist for learner assessments and investigate short-term and long-term skill retention rates.

## Conclusions

This study created and provided evidence of content validity and reliability for this checklist specific to this investigation using Lawshe’s methodology (Additional file 2: Figure S1). As such, this method of evaluating a checklist for validity and reliability evidence can be followed for other educational skills and processes in healthcare. This checklist can be used for teaching IV placement in healthcare students in the simulation procedural training lab.

## Additional file

Additional file 1: OSCE patient skills. (DOCX 29 kb)

Additional file 2: Figure S1. Inserting a peripheral intravenous (IV) line checklist. (DOCX 27 kb)

## Abbreviations

CFR: Code of Federal Register
CI: Confidence interval
CVI: Content validity index
CVR: Content validity ratio
G theory: Generalizability theory
IQR: Interquartile range
IV: Intravenous
