# Supplementary material for: Psychometric testing of a checklist for procedural training of peripheral intravenous insertion
Source: Adv Simul (Lond). 2019 Apr 18;4:5. doi: 10.1186/s41077-019-0092-y (PMC6471996; doi:10.1186/s41077-019-0092-y)
Supplement: Supplementary file 1 — OSCE patient skills. (DOCX 29 kb) [file 41077_2019_92_MOESM1_ESM.docx]

**OSCE Patient-Skills**

**IV Insertion**

**The items below require assessment as to whether the materials (the first eight items) and actions or skills are essential; useful, but not essential; or not necessary to routine IV insertion. Please indicate which category each material, action, or skill, in your expert opinion, belongs to. If a material, action, or skill is “useful, but not essential” or “not necessary” indicate why you believe so. For example,**

Is identifying where in town the patient lives

- essential?
- useful but not essential? Why? or
- not necessary? Why?

Why? (Required for all answers except “useful”.)

| Determining where in town a patient lives is not necessary because it has no bearing on |
| --- |
| IV insertion. Additionally, there are better ways to establish the identity of the patient |
| Such as MRN, DOB, and name. |

**Preparation and Preparation Materials**

Is Tegaderm^1–4^

- essential?
- useful but not essential? Why? or
- not necessary? Why?

Why? (Required for all answers except “useful”.)

|  |
| --- |
|  |
|  |

Is tape^1–4^

- essential?
- useful but not essential? Why? or
- not necessary? Why?

Why? (Required for all answers except “useful”.)

|  |
| --- |
|  |
|  |

Is an IV connector^1–4^

- essential?
- useful but not essential? Why? or
- not necessary? Why?

Why? (Required for all answers except “useful”.)

|  |
| --- |
|  |
|  |

Is a saline flush^1–4^

- essential?
- useful but not essential? Why? or
- not necessary? Why?

Why? (Required for all answers except “useful”.)

|  |
| --- |
|  |
|  |

Is the catheter^1–4^

- essential?
- useful but not essential? Why? or
- not necessary? Why?

Why? (Required for all answers except “useful”.)

|  |
| --- |
|  |
|  |

Are gloves^1–4^

- essential?
- useful but not essential? Why? or
- not necessary? Why?

Why? (Required for all answers except “useful”.)

|  |
| --- |
|  |
|  |

Is a tourniquet^1–4^

- essential?
- useful but not essential? Why? or
- not necessary? Why?

Why? (Required for all answers except “useful”.)

|  |
| --- |
|  |
|  |

**IV Insertion Skills and Actions**

Is gathering the preparation supplies^1–4^

- essential?
- useful but not essential? Why? or
- not necessary? Why?

Why? (Required for all answers except “useful”.)

|  |
| --- |
|  |
|  |

Is removing the heplock^5^

- essential?
- useful but not essential? Why? or
- not necessary? Why?

Why? (Required for all answers except “useful”.)

|  |
| --- |
|  |
|  |

Is attaching the extender (to the heplock)^5^

- essential?
- useful but not essential? Why? or
- not necessary? Why?

Why? (Required for all answers except “useful”.)

|  |
| --- |
|  |
|  |

Is flushing the extender^5^

- essential?
- useful but not essential? Why? or
- not necessary? Why?

Why? (Required for all answers except “useful”.)

|  |
| --- |
|  |
|  |

Is keeping the flush extender attached^5^

- essential?
- useful but not essential? Why? or
- not necessary? Why?

Why? (Required for all answers except “useful”.)

|  |
| --- |
|  |
|  |

Is donning gloves^1,4–6^

- essential?
- useful but not essential? Why? or
- not necessary? Why?

Why? (Required for all answers except “useful”.)

|  |
| --- |
|  |
|  |

Is identifying the non-dominant arm^5^

- essential?
- useful but not essential? Why? or
- not necessary? Why?

Why? (Required for all answers except “useful”.)

|  |
| --- |
|  |
|  |

Is placing the tourniquet proximal to insertion site tight enough to occlude venous flow^1,2,4,5^

- essential?
- useful but not essential? Why? or
- not necessary? Why?

Why? (Required for all answers except “useful”.)

|  |
| --- |
|  |
|  |

Is inspecting for veins that appear easier to cannulate distally on the non-dominant arm^1,4,5^

- essential?
- useful but not essential? Why? or
- not necessary? Why?

Why? (Required for all answers except “useful”.)

|  |
| --- |
|  |
|  |

Is palpating for veins that appear easier to cannulate distally on the non-dominant arm^1,4,5^

- essential?
- useful but not essential? Why? or
- not necessary? Why?

Why? (Required for all answers except “useful”.)

|  |
| --- |
|  |
|  |

Is cleaning the area circumferentially from center to periphery^1,2,4–6^

- essential?
- useful but not essential? Why? or
- not necessary? Why?

Why? (Required for all answers except “useful”.)

|  |
| --- |
|  |
|  |

Is not touching the intended cannulation site after it has been cleaned with alcohol^1,2,4–6^

- essential?
- useful but not essential? Why? or
- not necessary? Why?

Why? (Required for all answers except “useful”.)

|  |
| --- |
|  |
|  |

Is inserting the catheter bevel up^2,3,5^

- essential?
- useful but not essential? Why? or
- not necessary? Why?

Why? (Required for all answers except “useful”.)

|  |
| --- |
|  |
|  |

Is not touching the part of the catheter that will be inside the patient’s arm/vein^2,3,5^

- essential?
- useful but not essential? Why? or
- not necessary? Why?

Why? (Required for all answers except “useful”.)

|  |
| --- |
|  |
|  |

Is applying distal traction by placing your hand away from needle insertion site^1,3–6^

- essential?
- useful but not essential? Why? or
- not necessary? Why?

Why? (Required for all answers except “useful”.)

|  |
| --- |
|  |
|  |

Is advancing the needle at 30-45 degree angle with bevel facing up^1–5^

- essential?
- useful but not essential? Why? or
- not necessary? Why?

Why? (Required for all answers except “useful”.)

|  |
| --- |
|  |
|  |

When there is blood return, is lowering the angle of the needle to 10 degrees^1–5^

- essential?
- useful but not essential? Why? or
- not necessary? Why?

Why? (Required for all answers except “useful”.)

|  |
| --- |
|  |
|  |

Is advancing the catheter^1–5^ by extending the dominant index finger against the small tab that extends up from the top of the hub of the catheter, while maintaining slight traction on the vein with the non-dominant hand to prevent vein movement^2^

- essential?
- useful but not essential? Why? or
- not necessary? Why?

Why? (Required for all answers except “useful”.)

|  |
| --- |
|  |
|  |

Is retracting the needle essential^2,3,5^

- useful but not essential? Why? or
- not necessary? Why?

Why? (Required for all answers except “useful”.)

|  |
| --- |
|  |
|  |

Is removing the needle essential^2,3,5^

- useful but not essential? Why? or
- not necessary? Why?

Why? (Required for all answers except “useful”.)

|  |
| --- |
|  |
|  |

Is applying pressure above the IV site without letting go of the catheter after the needle is removed^4^

- essential?
- useful but not essential? Why? or
- not necessary? Why?

Why? (Required for all answers except “useful”.)

|  |
| --- |
|  |
|  |

Is removing the tourniquet^1–5^

- essential?
- useful but not essential? Why? or
- not necessary? Why?

Why? (Required for all answers except “useful”.)

|  |
| --- |
|  |
|  |

Is connecting an IV tube extender^1–5^

- essential?
- useful but not essential? Why? or
- not necessary? Why?

Why? (Required for all answers except “useful”.)

|  |
| --- |
|  |
|  |

Is drawing back on the plunger to check for blood return^4^

- essential?
- useful but not essential? Why? or
- not necessary? Why?

Why? (Required for all answers except “useful”.)

|  |
| --- |
|  |
|  |

Is locking the extender securely proximal to the catheter hub^4^

- essential?
- useful but not essential? Why? or
- not necessary? Why?

Why? (Required for all answers except “useful”.)

|  |
| --- |
|  |
|  |

Is removing the syringe^4^

- essential?
- useful but not essential? Why? or
- not necessary? Why?

Why? (Required for all answers except “useful”.)

|  |
| --- |
|  |
|  |

Is reconnecting the heplock^3^

- essential?
- useful but not essential? Why? or
- not necessary? Why?

Why? (Required for all answers except “useful”.)

|  |
| --- |
|  |
|  |

Is applying Tegaderm over the IV insertion site on the hub of the catheter^1–4,6^

- essential?
- useful but not essential? Why? or
- not necessary? Why?

Why? (Required for all answers except “useful”.)

|  |
| --- |
|  |
|  |

Is taping the catheter and tubing in place with two separate pieces of tape^1,4^

- essential?
- useful but not essential? Why? or
- not necessary? Why?

Why? (Required for all answers except “useful”.)

|  |
| --- |
|  |
|  |

Is disposing of the needle in a proper sharps container^1,4^

- essential?
- useful but not essential? Why? or
- not necessary? Why?

Why? (Required for all answers except “useful”.)

|  |
| --- |
|  |
|  |

References

1. Tintinalli JE, Stapczynski JS, Ma OJ, Yealy DM, Meckler GD, Cline DM. *Tintinalli’s Emergency Medicine: A Comprehensive Study Guide*. Vol 8th ed. Columbus, OH: McGraw-Hill Education / Medical; 2016.

2. Frank RL. Peripheral venous access in adults. *UpToDate*. 2016.

3. Leuven W& Van, Wilkinson & Van Leuven. *Procedure Checklist Chapter 36: Initiating a Peripheral Intravenous Infusion*.; 2007.

4. Ross University School of Medicine. Intravenous Cannulation Checklist. 2016.

5. Learning TD. *Skill 18: Insertion of a Peripheral IV Line*.

6. Infusion Nursing Society. Short Peripheral Catheter Checklist: Think Safety, Insert Safely. *Infus Nurs Soc*. 2014;(1):1. doi:10.1007/s13398-014-0173-7.2.
