# Supplementary material for: Psychometric testing of a checklist for procedural training of peripheral intravenous insertion
Source: Adv Simul (Lond). 2019 Apr 18;4:5. doi: 10.1186/s41077-019-0092-y (PMC6471996; doi:10.1186/s41077-019-0092-y)
Supplement: Supplementary file 2 — Figure S1. Inserting a peripheral intravenous (IV) line checklist. (DOCX 27 kb) [file 41077_2019_92_MOESM2_ESM.docx]

**Figure S1. Inserting a Peripheral Intravenous (IV) Line Checklist**

| **Name:** |  | **Date:** |  |
| --- | --- | --- | --- |

|  | **Item** | | | | | | | | | **No** | **Yes** |
| --- | --- | --- | --- | --- | --- | --- | --- | --- | --- | --- | --- |
| 1. | Did the learner collect and correctly prepare all the supplies? | | | | | | | | |  |  |
|  | Transparent Medical Dressing | |  | Saline flush |  | IV catheter needle |  | IV extension tubing |  |  |  |
|  | Tourniquet | |  | Antiseptic |  | Luer adapter/cap |  | Gloves |  |  |  |
| 2. | Did the learner don gloves? | | | | | | | | |  |  |
| 3. | Did the learner identify the nondominant arm? | | | | | | | | |  |  |
| 4. | Did the learner flush the IV extension tubing? | | | | | | | | |  |  |
| 5. | Did the learner attach the Luer adapter/cap to the end of the IV extension tubing? | | | | | | | | |  |  |
| 6. | Did the learner inspect the nondominant arm for the most distal veins that appear easiest to cannulate? | | | | | | | | |  |  |
| 7. | Did the learner prepare the skin with an antiseptic (70% alcohol, tincture of iodine, an iodophor or chlorhexidine gluconate) before peripheral venous catheter insertion? | | | | | | | | |  |  |
| 8. | Did the learner refrain from touching the intended cannulation site after it has been cleaned? | | | | | | | | |  |  |
| 9. | Did the learner advance the needle into the vein at 30^o^-40^o^ angle with the bevel facing up? | | | | | | | | |  |  |
| 10. | Did the learner advance the catheter by extending the dominant index finger? | | | | | | | | |  |  |
| 11. | Did the learner retract the needle? | | | | | | | | |  |  |
| 12. | Did the learner remove the needle? | | | | | | | | |  |  |
| 13. | Did the learner apply pressure above the IV site without letting go of the catheter after the needle is removed? | | | | | | | | |  |  |
| 14. | Did the learner lock the extender securely proximal to the catheter hub? | | | | | | | | |  |  |
| 15. | Did the learner remove the saline flush syringe? | | | | | | | | |  |  |
| 16. | Did the learner apply transparent medical dressing over the IV insertion site on the hub of the catheter? | | | | | | | | |  |  |
| **Instructor:** | |  | | | | |  |  |  |  |  |
